# Inhibitory effect of fucoidan on TNF-α-induced inflammation in human retinal pigment epithelium cells

**DOI:** 10.3389/fnut.2023.1162934

**Published:** 2023-04-12

**Authors:** Sol Lee, Eun Jeoung Lee, Gyu Min Lee, Ji-Hyun Yun, Wonbeak Yoo

**Affiliations:** ^1^AceBiome Inc., Seoul, Republic of Korea; ^2^R&D Center, AceBiome Inc., Daejeon, Republic of Korea

**Keywords:** *Sargassum horneri*, AB_SH, fucoidan, anti-inflammation, NF-κB/MAPK, ARPE-19

## Abstract

*Sargassum horneri* (*S. horneri*) is a brown seaweed that contains a fucose-rich sulfated polysaccharide called fucoidan and is known to possess beneficial bioactivities, such as anti-inflammatory, antiviral, antioxidative, and antitumoral effects. This study aimed to determine the anti-inflammatory effects of AB_SH (hydrothermal extracts from *S. horneri*) and its bioactive compound (fucoidan) against tumor necrosis factor alpha (TNF-α)-induced inflammation in human retinal pigment epithelial (RPE) cells. AB_SH did not exhibit any cytotoxicity, and it decreased the mRNA expression of interleukin (IL)-6 and IL-8 and the production of the cytokines IL-6 and TNF-α. It also suppressed the expression levels of phosphorylated nuclear factor kappa B (NF-κB) and mitogen-activated protein kinases (MAPKs), including c-Jun amino-terminal kinases (JNK), p38 protein kinases (p38), and extracellular signal-regulated kinase (ERK) proteins, suggesting that AB_SH inhibits activation of the NF-kB/MAPK signaling pathway. Since fucoidan was identified in the composition analysis of AB_SH, it was additionally shown to be required for its anti-inflammatory effects in TNF-α-stimulated human RPE cells. In line with the AB_SH results, fucoidan reduced the mRNA levels of IL-6, IL-1ß, and IL-8 and production of the cytokines IL-6, TNF-α, and IL-8 through the downregulation of the NF-kB/MAPK signaling pathway in a dose-dependent manner. Collectively, the ability of AB_SH from *S. horneri* hydrothermal extracts to reduce inflammation indicates that it may be a good functional ingredient for managing ocular disorders.

## Introduction

Retinal pigment epithelium (RPE) is a monolayer of pigment cells that maintains photoreceptor integrity, primarily by phagocytosing and recycling retinal photoreceptor outer segments (1). In addition, RPE cells are involved in immune responses by maintaining immune homeostasis within the eye (2). Thus, inflammatory dysfunction of RPE cells can lead to various pathological conditions including age-related macular degeneration (AMD), proliferative vitreoretinopathy (PVR), and diabetic retinopathy (DR) (1, 3). Ocular inflammation has been suggested to involve the secretion of inflammatory mediators by RPE cells. Some RPE-derived pro-inflammatory cytokines include interleukin (IL)-1, IL-6, TNF-α, and chemokines such as monocyte chemotactic protein (MCP)-1 and IL-8 (1, 4). In particular, the inflammatory cytokine TNF-α is one of the key regulators of inflammatory responses and has been reported to be involved in the pathogenesis of a number of inflammatory diseases (5). TNF-α is involved in the transcription of genes that regulate inflammation, cell survival, proliferation, and differentiation through activation of the NF-κB pathway (6). Therefore, regulating the TNF-α-induced inflammatory response may be key to overcoming the dysfunction of RPE cells.

*Sargassum horneri* (*S. horneri*) is a brown algae that grows on the coast of East Asia and has been reported to have medicinal benefits, such as anti-oxidant, neuroinflammatory, and anti-inflammatory effects (7–11). *S. horneri* contains high concentrations of polysaccharides such as fucoidan, which mainly consists of L-fucose and sulfate ester groups (12, 13). Fucoidan has been reported to exhibit a variety of therapeutic benefits, including anticancer, immune-regulatory, antiviral, anti-obesity, and anti-inflammatory effects, both *in vitro* and in animal studies (14–18). The most potent mechanism of fucoidan is downregulation of and mitogen-activated protein kinases (MAPKs) and NF-κB signaling pathways and reduction of pro-inflammatory cytokine production (14).

In the present study, we aimed to evaluate *S. horneri* inhibition of the inflammatory response of ARPE-19 cells stimulated by TNF-α, and the potential of its active compound, fucoidan, in eye health by elucidation of the underlying mechanism.

## Materials and methods

### Chemicals and reagents

All chemicals and reagents were obtained from Sigma-Aldrich (St. Louis, MO, USA) unless stated otherwise. TNF-α was purchased from PeproTech (Rocky Hill, NJ, USA). TRIzol^®^ reagent and a bicinchoninic acid (BCA) protein assay kit were purchased from Thermo Fisher Scientific (Waltham, MA, USA). The MAPK Family Antibody Sampler Kit (cat. no. 9926), and a phospho-MAPK Family Antibody Sampler Kit (cat. no. 9910) were purchased from Cell Signaling Technology (Danvers, MA, USA). Sugar standards (mannose, rhamnose, glucuronic acid, glucose, galactose, xylose, and fucose), trifluoroacetic acid, sodium hydroxide, 3-methyl-1-phenyl-2-pyrazoline-5-one (PMP), hydrochloric acid, and ammonium acetate were purchased from Sigma-Aldrich (USA). The InfinityLab Poroshell 120 120 EC-C18 was purchased from Agilent. Methanol and acetonitrile were obtained from Baker Chemical Company (Phillipsburg, NJ, USA). Chloroform was obtained from Duksan Pure Chemicals Co. (Ansan-si, Republic of Korea). All solvents used for chromatography were high-performance liquid chromatography (HPLC) grade.

### Preparation of AB_SH from *S. horneri* and composition analysis

**FIGURE 1:**
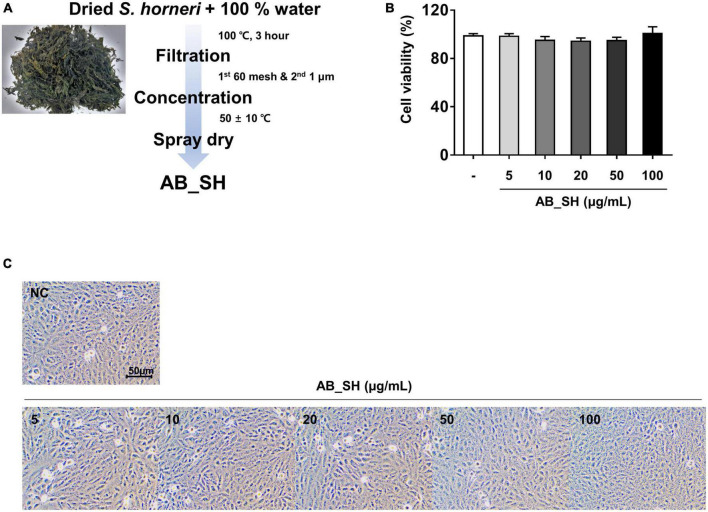
Cell viability and cell morphology following AB_SH treatment of ARPE-19 cells. **(A)** Schematic diagram for the extraction process for AB_SH. ARPE-19 cells were treated with various concentrations of AB_SH. **(B)** cell viability and **(C)** cell morphology after treatment (5, 10, 20, 50, or 100 μg/mL) for 24 h. The values represent the mean ± SD of three independent experiments.

AB_SH (a powder of hydrothermal extract from *S. horneri* collected during the spring season in 2021 along the coast of Jeju Island in South Korea) was obtained from Ohta Foods Korea Co., Ltd. (Jeollanam-do, Republic of Korea). Briefly, water extraction was performed at 100°C for 3 h. The extract was further filtered sequentially (60 mesh and 1 micron mesh) and concentrated by rotary vacuum evaporator (50 ± 10°C). Finally, it was spray-dried to obtain powdered *S. horneri* (AB_SH) and used for further analysis (Figure 1A). General and carbohydrate analysis of AB_SH was performed by Humanbio Co. Ltd. as a system certified in Korea according to the Ministry of Food and Drug Safety Notice No. 2021-26 (full text of notification of standards and specifications of food)^1^. Humanbio Co. Ltd. has been designated as a food (functional food for health) inspection agency by the Korea System Certification Body and Daejeon Regional Office of Food and Drug Safety (Korea).

### Preparation of 3-methyl-1-phenyl-5-pyrazolone (PMP)-derivatives and chromatography

AB_SH was hydrolyzed as described by Fu et al. (19). Briefly, 10 mL of 2 M trifluoroacetic acid (TFA) was added to 0.1 g of AB_SH and heated at 100°C for 120 min. The mixture was then cooled to room temperature. After hydrolysis, the sample was filtered and adjusted to pH 7 using 2 M sodium hydroxide (NaOH). This was further used in the PMP-derivative reaction. The hydrolyzed AB_SH and PMP-derivative reactions were performed according to the method described by Honda et al. (20). Hydrolyzed AB_SH samples (2 mL) were mixed with 2 mL of 0.5 M PMP-MeOH solution and with 2 mL of 0.3 M NaOH solution, reacted in a water bath at 70°C for 30 min, and then cooled to room temperature. Next, 2 mL of 0.3 M HCl was added to the mixture and stirred for 1 min until the formation of precipitates. These were mixed with 4 mL of chloroform at 20°C, and the aqueous layer was filtered through a 0.45 μm PTFE membrane syringe filter (Whatman International, Maidstone, UK). PMP-labeled samples were analyzed on an Agilent 1,260 Infinity II System coupled with a photodiode array detector (Agilent, Santa Clara, CA, USA). The analytical column was an InfinityLab Poroshell 120 EC-C18 column (150 × 4.6 mm, 2.7 μm) operated at 35°C. The injection volume was 10 μL and elution was performed at a flow rate of 1 mL/min. Fucose was detected using a PDA detector (Agilent) at a wavelength of 254 nm. The mobile phase A consisted of acetonitrile and mobile phase B was 100 mM ammonium acetate buffer, using gradient elution as described in Table 1. Sugar standard solutions were prepared according to the same steps described above.

**TABLE 1:** Gradient elution program.

Time (min)	(A)	(B)
Initial	10	90
5	10	90
6	20	80
40	20	80
41	10	90
51	10	90

The mobile phase consisted of (A) 100% acetonitrile and (B) 10 mM ammonium acetate.

### Cell culture

The ARPE-19 cells were grown in a 1:1 mixture of Dulbecco’s modified Eagle’s medium/Ham’s F-12 Nutrient Mixture (Welgene, Daegu, Korea) supplemented with 10% fetal bovine serum (HyClone™, Logan, UT, USA) and antibiotic/antimycotic solution (HyClone™) at 37°C and in an atmosphere containing 5% CO_2_.

### Cell viability assay

Cell viability was measured using a thiazolyl blue tetrazolium bromide (MTT) assay. The MTT powder was obtained from Tokyo Chemical Industry Co., Ltd. (Tokyo, Japan). ARPE-19 cells were plated in 24-well plates at a density of 5 × 10^4^ cells/well for 24 h. After incubation, AB_SH (5, 10, 20, 50, and 100 μg/mL) or fucoidan (1, 5, 10, and 50 μg/mL) was added at the indicated concentrations and was incubated for 24 h at 37°C in humidified air and 5% CO_2_. Concentration of AB_SH and fucoidan were referred to previous studies (21, 22). A stock solution of MTT (5 mg/mL in PBS) was added to each well plate at a final concentration of 0.5 mg/mL according to the manufacturer’s protocol. After incubation for 2 h at 37°C, the formazan was solubilized in dimethyl sulfoxide (DMSO). The cell viability was then measured using a Multiskan™ SkyHigh microplate reader (Thermo Fisher Scientific) at 590 nm. The percentage of cells exhibiting cytotoxicity was determined relative to that of the control group.

### RNA preparation and quantitative real-time PCR

For mRNA expression analysis, ARPE-19 cells (1 × 10^5^ cells/well) were seeded in 12-well plates and incubated. After incubation, ARPE-19 cells were pretreated with the indicated concentrations of AB_SH (10 and 100 μg/mL) or fucoidan (10 and 50 μg/mL) for 1 h and then incubated with 50 ng/mL TNF-α for 1 h. The cells were then harvested, and total RNA was extracted with TRIzol^®^ reagent (Thermo Fisher Scientific), according to the manufacturer’s protocol. The isolated RNA concentration was measured using a OPTIZEN NanoQ Lite (KLAB, Daejeon, Korea). Complementary DNA (cDNA) is (1 μg) was synthesized using a RevertAid RT Reverse Transcription Kit (Thermo Fisher Scientific). Quantitative real-time PCR (real-time qPCR) was performed using AccuPower^®^ 2X GreenStar™ qPCR Master Mix (Bioneer, Daejeon, Korea), according to the manufacturer’s instructions. Each 20 μL real-time qPCR reaction contained an amount of cDNA equivalent to 1 μL of total RNA (20 ng), 1 μL of the forward and reverse primer each (10 μM), 10 μL of the 2X GreenStar Master Mix, and 7 μL of RNase-free water. 18S rRNA was used as a reference gene for the normalization of all samples, and the gene expression levels were calculated using a previously reported method (23). All primers used are listed in Supplementary Table 1.

### Measuring cytokine and chemokine production

The amount of secreted cytokines in cell culture supernatants was determined using an enzyme-linked immunosorbent assay (ELISA). ARPE-19 cells were seeded into 12-well plates (1 × 10^5^ cells/well) and incubated for 24 h. After incubation, the cells were pre-treated with the indicated concentrations of AB_SH or fucoidan for 1 h and then incubated with 50 ng/mL TNF-α for 24 h. IL-6 (cat. no. ab178013), TNF-α (cat. no. ab181421), and IL-8 (cat. no. ab214030) in the supernatants were measured using an ELISA kit according to the manufacturer’s recommendations. All ELISA kits were purchased from Abcam (Cambridge, UK).

### Western blot analysis

ARPE-19 cells were seeded in a 60 mm dish at a density of 6 × 10^5^ cells/well and incubated for 24 h. After incubation, the cells were exposed to fresh culture media containing AB_SH or fucoidan for 1 h and then stimulated with TNF-α (50 ng/mL) for 30 min or 3 h. The cells were washed with cold PBS and lysed using radioimmunoprecipitation assay (RIPA) buffer containing a protease and phosphatase inhibitor cocktail. The concentration of the extracted proteins was measured using a BCA protein assay kit (Thermo Fisher Scientific) according to the manufacturer’s protocol. The extracted proteins (20 μg) were separated by sodium dodecyl sulfate-polyacrylamide gel electrophoresis (SDS-PAGE) on an 8% gel and then transferred to polyvinylidene difluoride (PVDF) membranes (Thermo Fisher Scientific). Phosphorylated protein blots were blocked with 3% BSA for 1 h and total protein blots were blocked with 5% skim milk for 1 h. and then the membranes were incubated with the following primary antibodies against p-NF-κB (Invitrogen, Eugene, OR, USA), NF-κB, p-JNK, JNK, p-p38, p38, p-ERK, and ERK (Cell Signaling Technology, Inc., Beverly, MA, USA) at 4°C overnight. After incubation, horseradish peroxidase (HRP)-conjugated secondary antibodies were added to the membranes. After 1 h, the blots were washed and the protein bands detected using SuperSignal™ West Pico PLUS Chemiluminescent Substrate (Thermo Fisher Scientific) and photographed with an iBright™ CL750 Imaging System (Thermo Fisher Scientific). GAPDH was used as the protein loading control.

### Statistical analysis

All data were analyzed using GraphPad Prism 7.0 (San Diego, CA, USA). The data are expressed as mean values with their standard errors. The statistical analyses were performed using Student’s *t*-test or one-way analysis of variance for multiple comparisons. For all comparisons, a *p*-value < 0.05 was considered statistically significant.

## Results

### Effect of AB_SH on ARPE-19 cell viability

The preparation process of AB_SH from *S. horneri* is shown in Figure 1A. To determine whether AB_SH exerted toxicity toward ARPE-19 cells, we determined the cell viability by MTT assay. As shown in Figure 1B, there was no significant difference between different concentrations of AB_SH (5–100 μg/mL). Consistent with this result, phase-contrast microscopy also revealed that there were no significant changes in cellular morphology (Figure 1C). Therefore, AB_SH was used at concentrations of 5–100 μg/mL to investigate the anti-inflammatory effects of TNF-α in ARPE-19 cells.

### Anti-inflammatory effect of AB_SH on TNF-α induced inflammation in ARPE-19 cells

**FIGURE 2:**
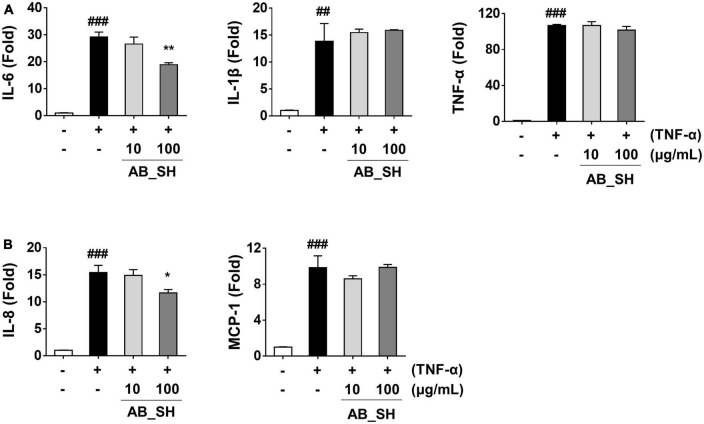
Effect of AB_SH on mRNA expression of cytokines and chemokines in TNF-α-stimulated ARPE-19 cells. ARPE-19 cells were pre-treated with AB_SH for 1 h and subsequently stimulated with 50 ng/mL TNF-α for 1 h. The mRNA expression was analyzed by real-time RT-PCR. mRNA expression levels of **(A)** the cytokines IL-6, IL-1β, and TNF-α and **(B)** the chemokines IL-8 and MCP-1. The values represent the mean ± SD of three independent experiments. ^##^*p* < 0.01 and ^###^*p* < 0.001 compared with the control. *p < 0.05 and **p < 0.01 compared with the TNF-α-treated control, respectively.

**FIGURE 3:**
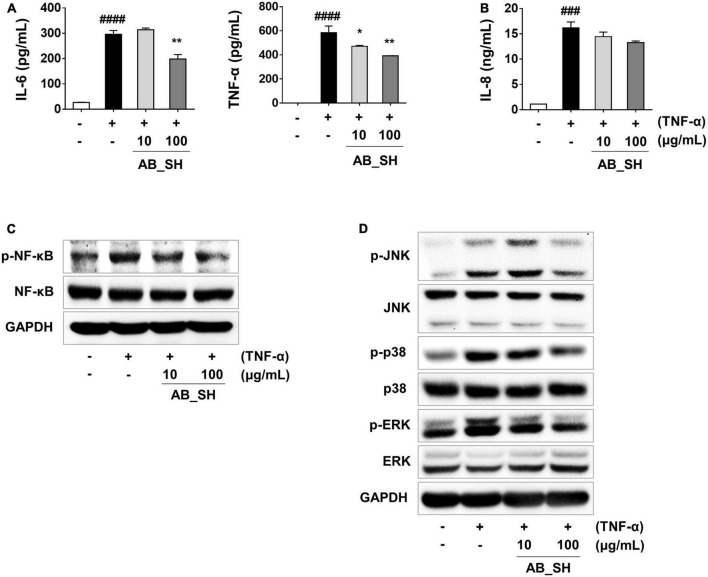
Anti-inflammatory effect of AB_SH on cytokine production and NF-κB/MAPK signaling in TNF-α-stimulated ARPE-19 cells. ARPE-19 cells were pre-treated with AB_SH for 1 h and then stimulated with 50 ng/mL TNF-α for 24 h. The levels of cytokines and chemokines were measured by ELISA. **(A)** Levels of the cytokines IL-6 and TNF-α, and **(B)** level of the chemokine IL-8. Effect of AB_SH on **(C)** NF-κB and **(D)** MAPK signaling pathways. The cells were pre-treated with AB_SH for 1 h and then stimulated with 50 ng/mL TNF-α for 30 min. The cell lysates were subjected to western blot analysis. GAPDH was used as a loading control. The values represent the mean ± SD of three independent experiments. ^###^*p* < 0.001, ^####^*p* < 0.0001 compared with the control. *p < 0.05 and **p < 0.01 compared with the TNF-α-treated control, respectively.

To determine whether pretreatment with AB_SH in the presence of TNF-α resulted in an anti-inflammatory effect in ARPE-19 cells, we first performed RT-PCR to detect mRNA expression, which has been implicated in cellular inflammation. The results revealed that all inflammation-related cytokines and chemokines were significantly increased after treatment with TNF-α, whereas the levels of IL-6 and IL-8 mRNA were significantly reduced after treatment with AB_SH at 100 μg/mL (Figures 2A, B). In addition, the levels of cytokines and chemokines involved in inflammation were analyzed using ELISA. As shown in Figure 3A, the production of IL-6 and TNF-α was significantly decreased after treatment with AB_SH at a dose of 100 μg/mL, whereas IL-8 tended to decrease albeit not in a statistically significant manner (Figure 3B). To confirm the anti-inflammatory effect of AB_SH on TNF-α-induced inflammatory signaling in ARPE-19 cells, the phosphorylation of NF-κB and MAPK signaling pathway components, including JNK, p38, and ERK, was investigated by western blot analysis. As shown in Figure 3C, TNF-α treatment enhanced the phosphorylation of NF-κB, which was significantly attenuated by AB_SH treatment. Collectively, AB_SH had an anti-inflammatory effect in TNF-α-induced ARPE-19 cells and was related to the downregulation of NF-κB/MAPK signaling pathways.

### Composition analysis of AB_SH

**FIGURE 4:**
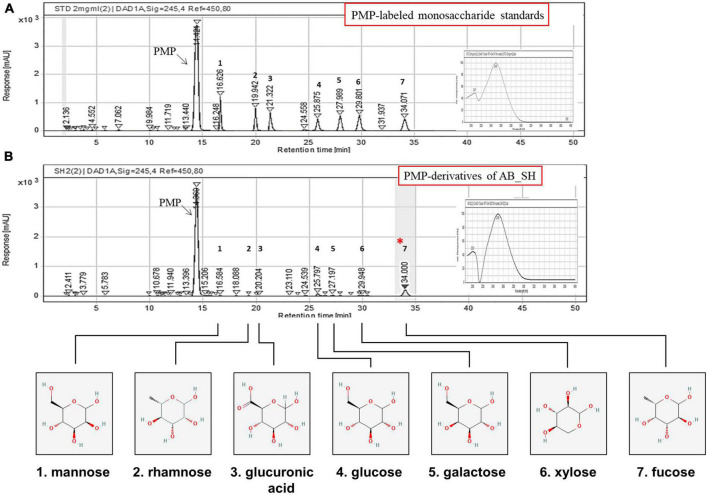
Composition analysis and structures of the major polysaccharides in AB_SH. Chromatograms of **(A)** PMP-labeled sugar standards and **(B)** PMP-derivatives of acid hydrolysates of AB_SH.

The proximate nutritional composition results are presented in Table 2. The main component of AB_SH was carbohydrates (46.87 g/100 g), and further characterization of AB_SH revelated that the carbohydrate composition in AB_SH mainly consisted of mannose, rhamnose, glucuronic acid, glucose, galactose, xylose, and fucose at 2.48, 2.13, 0.94, 7.92, 4.49, 4.20, and 77.84%, respectively, (Table 3). In recent studies, Hong et al. (24) and Filippo-Herrera et al. (25) suggested that fucoidan is a major component of brown algae, including *S. horneri*, and is composed of seven monosaccharides from hydrothermal extracts of *S. horneri*, mainly fucose. In light of these results, we conducted a composition analysis, and AB_SH was found to also be composed of the same monosaccharides, and the fucose content was the highest (Figures 4A, B and Table 3). Therefore, fucoidan was considered the main substance in AB_SH and was used for further analyses of the anti-inflammatory effects on TNF-α-induced inflammation in ARPE-19 cells.

**TABLE 2:** Nutrition content of AB_SH.

Composition	Content (g/100 g)
Carbohydrate	46.87 ± 8.44
Protein	10.46 ± 1.91
Sugar	0.45 ± 0.63
Saturated fat	0.02 ± 0.01
Trans fat	0.01 ± 0.01
Moisture, Ash, Crude fiber, etc.,	42.19 ± 7.59

**TABLE 3:** Carbohydrate composition analysis of AB_SH.

Composition	Content (%)
Mannose (1)	2.48
Rhamnose (2)	2.13
Glucuronic acid (3)	0.94
Glucose (4)	7.92
Galactose (5)	4.49
Xylose (6)	4.2
Fucose (7)	77.84

### Effect of fucoidan on ARPE-19 cell viability

**FIGURE 5:**
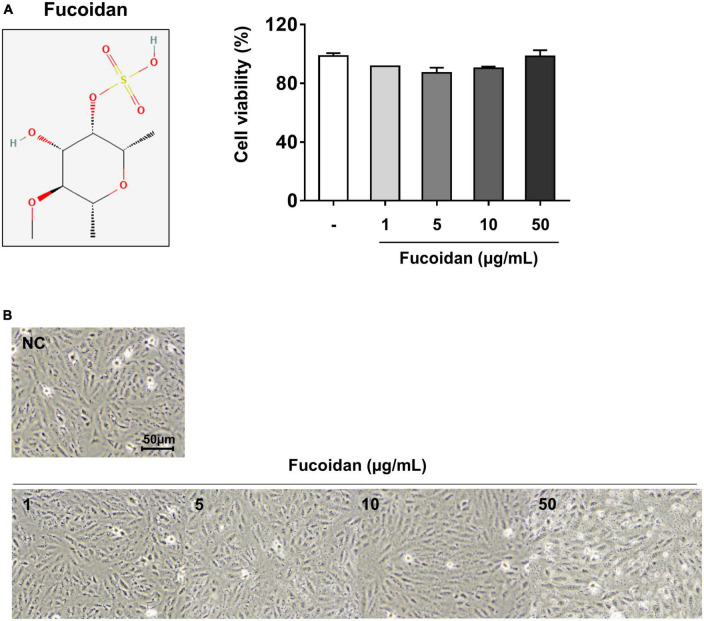
Cell viability and morphology of fucoidan-treated ARPE-19 cells. ARPE-19 cells were treated with various fucoidan concentrations. **(A)** Cell viability and **(B)** cell morphology after treatment (1, 5, 10, or 50 μg/mL) for 24 h. The values represent the mean ± SD of three independent experiments.

The cytotoxicity of fucoidan was determined by measuring cell viability by MTT assay. As shown in Figure 5A, fucoidan had no effect on cell viability at different concentrations (1–50 μg/mL). In addition, the cell morphology by phase-contrast microscopy was not changed (Figure 5B). Therefore, fucoidan at a concentration of 10–50 μg/mL was used to investigate the anti-inflammatory effects of TNF-α in ARPE-19 cells.

### Anti-inflammatory effect of fucoidan on TNF-α-induced inflammation in ARPE-19 cells

**FIGURE 6:**
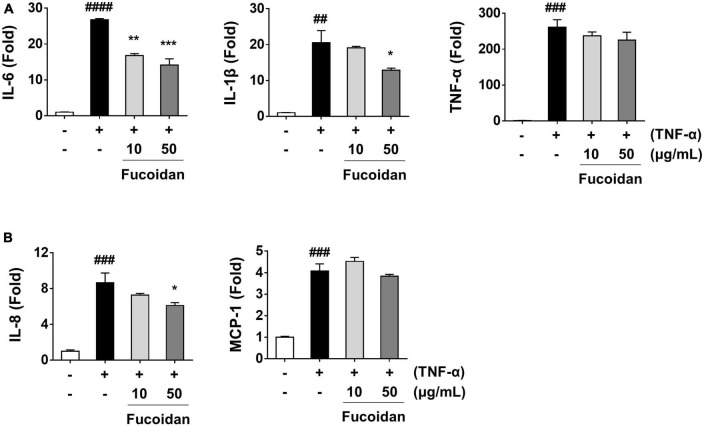
Effect of fucoidan on mRNA expression of pro-inflammatory mediators in TNF-α-stimulated ARPE-19 cells. ARPE-19 cells were pre-treated with fucoidan for 1 h and then stimulated with 50 ng/mL TNF-α for 1 h. The mRNA expression was analyzed by real-time RT-PCR. mRNA expression levels of **(A)** the cytokines IL-6, IL-1β, and TNF-α and **(B)** the chemokines IL-8 and MCP-1. The values represent the mean ± SD of three independent experiments. ^##^*p* < 0.01, ^###^*p* < 0.001 and ^####^*p* < 0.0001 compared with the control. *p < 0.05, **p < 0.01 and ***p < 0.001 compared with the TNF-α-treated control, respectively.

**FIGURE 7:**
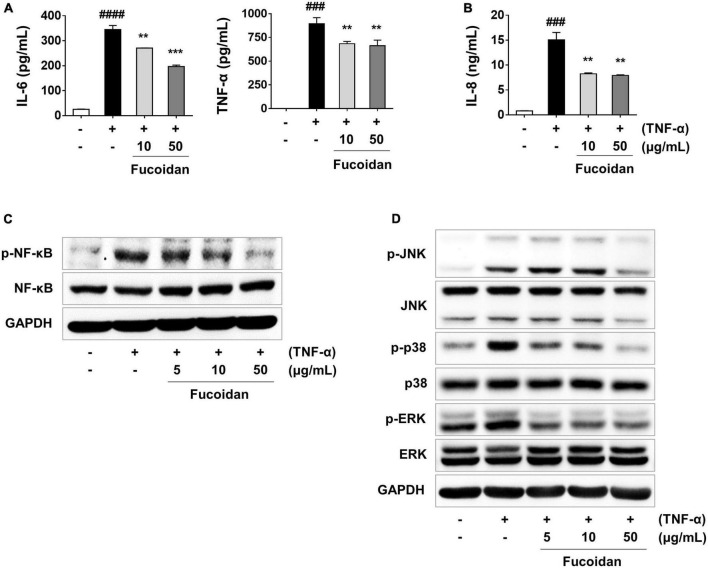
Anti-inflammatory effect of fucoidan on cytokine production and NF-κB/MAPK signaling in TNF-α-stimulated ARPE-19 cells. ARPE-19 cells were pre-treated with fucoidan for 1 h and then stimulated with 50 ng/mL of TNF-α for 24 h. **(A)** Levels of the cytokines IL-6 and TNF-α, and **(B)** level of the chemokine IL-8. The production of cytokines and chemokines was measured by ELISA. Effect of fucoidan on NF-κB and MAPK signaling pathways. **(C)** Western blotting for NF-κB in cells pre-treated with fucoidan for 3 h and then stimulated with 50 ng/mL of TNF-α for an additional 30 min. **(D)** Western blotting for MAPKs in cells pre-treated with fucoidan for 30 min and then stimulated with 50 ng/mL of TNF-α for 30 min. GAPDH was used as a loading control. The values represent the mean ± SD of three independent experiments. ^###^*p* < 0.001, ^####^*p* < 0.0001 compared with the control. **p < 0.01 and ***p < 0.001 compared with the TNF-α-treated control, respectively.

The anti-inflammatory effects of fucoidan on inflammation-related cytokine and chemokine mRNA levels were analyzed in ARPE-19 cells stimulated with TNF-α. As shown in Figure 6A, pretreatment of ARPE-19 cells with fucoidan significantly decreased the mRNA expression of IL-6 and IL-1ß in TNF-α-induced inflammation. Under the same conditions, the mRNA level of the chemotactic cytokine IL-8 was reduced after treatment with fucoidan (Figure 6B), whereas the mRNA levels of TNF-α and MCP-1 were not changed. To better understand the inhibitory effects of fucoidan on TNF-α-induced inflammation, the production of pro-inflammatory cytokines and chemokines was investigated by ELISA in ARPE-19 cells. The results revealed that pretreatment of TNF-α-treated ARPE-19 cells with fucoidan decreased the levels of IL-6, TNF-α, and IL-8 in a dose-dependent manner (Figures 7A, B). Notably, pretreatment with fucoidan in TNF-α-treated ARPE-19 cells resulted in a dose-dependent manner decrease in the levels of phosphorylated NF-κB and MAPK, including JNK, p38, and ERK, (Figures 7C, D). These results indicate that fucoidan exerts anti-inflammatory effects by inhibiting the NF-κB/MAPK signaling pathway in ARPE-19 cells, as confirmed by the AB_SH results.

## Discussion

Ocular disorders, such as inflammation of the posterior eye segment, can cause damage resulting in visual impairment, including AMD, DR, retinitis pigmentosa, and staphylitis (26). Previous studies have reported that inflammation may play a key role in RPE dysfunction, degeneration, and loss in retinal disease (27, 28). Accordingly, TNF-α appears to play a major role in the ophthalmic pathogenesis of the etiology, and activation of the TNF-α signaling pathway initiates the downstream induction of MAPK via phosphorylation of transcription factors such as NF-κB (6, 29, 30). Their roles have been studied in the occurrence of retinal disease, including IL-1ß, IL-6, IL-8, and MCP-1, which are the most commonly investigated pro-inflammatory cytokines in ophthalmic studies (31–33). Moreover, there is increasing evidence that inhibition of TNF-α can be an option for the treatment of eye diseases (34). However, TNF-α inhibitors can have systemic side effects that are related to malignancy, serious infections, retinal vein occlusion (RVO), inflammation, and demyelination (35–37), and numerous attempts have been made to evaluate their anti-inflammatory effects as a functional ingredient of natural substances that were previously determined to be safe and easy to use (38–40). In our study, we found that TNF-α stimulation led to the upregulation of IL-6, IL-1ß, IL-8, and TNF-α, while treatment with AB_SH and fucoidan inhibited mRNA and cytokine production in AREP-19 cells. Treatment with AB_SH and fucoidan also decreased the phosphorylation of NF-κB/MAPKs in these cells. These results indicated that AB_SH and its bioactive compound fucoidan protected cells from TNF-α-stimulated inflammation.

AB_SH was obtained from hydrothermal extracts of *S. horneri* and mainly included carbohydrates (Figure 1A and Table 2). Natural products of brown algae are known to help relieve various diseases, such as inflammation, oxidative stress, and diabetes (41–43). *Sargassum horneri* (*S. horneri*), a large proportion of brown algae, is consumed as a functional and medicinal food because of its abundance of bioactive compounds, including sulfated polysaccharides and phlorotannins (44). According to previous studies, sulfated polysaccharides exert strong anti-inflammatory, antiviral, anti-oxidant, and immunostimulatory effects (45–49). Since much of *S. horneri*’s medicinal influence is attributed to the activity of its sulfated polysaccharides, fucoidan extracted from the brown algae including *S. horneri* can act as prebiotics by modulating the abundance and diversity of gut microbiota (11). Therefore, fucose-rich sulfated polysaccharides, called fucoidan, are considered natural bioactive ingredient for functional foods (50, 51). In the present study, AB_SH derived from *S. horneri* was composed of seven monosaccharides, which are the main components of fucoidan, and it was identified by component analysis (Figure 4 and Table 3). We also found that the fucose content in AB_SH was the highest amount of total carbohydrate, which is the main fucoidan substance, as previously reported s (24, 25). Therefore, we considered that fucoidan is the main bioactive compound in AB_SH and hence studied it further.

In this study, we demonstrated that AB_SH significantly reduced the mRNA expression of TNF-α-induced IL-6 and IL-8 in ARPE-19 cells and decreased the production of IL-6 and TNF-α in cells stimulated with TNF-α. Western blot analysis showed that AB_SH suppressed the TNF-α-stimulated phosphorylation of NF-κB/MAPKs (Figures 2, 3). By component analysis of AB_SH, fucoidan contained high levels of seven monosaccharides (25). In addition to documentation of the anti-inflammatory effects of AB_SH, fucoidan was shown to be the main active component. The results indicate that fucoidan treatment reduced the mRNA expression of IL-6, IL-1ß, and IL-8 in ARPE-19 cells (Figure 6). ELISA showed that the production of IL-6, TNF-α, and IL-8 decreased after treatment with fucoidan in TNF-α-stimulated cells. Furthermore, fucoidan treatment inhibited the TNF-α-stimulated phosphorylation of NF-κB/MAPKs, as shown by western blot analysis (Figure 7). As the present study used an *in vitro* RPE model, it remains to be determined whether AB_SH and fucoidan have anti-inflammatory effects on primary and inflamed RPE cells *in vivo*. Further studies are required to investigate the relationship between the anti-inflammatory potential of AB_SH and fucoidan and the disease model using AB_SH to further verify the results of the current study.

## Conclusion

In summary, our findings indicate that AB_SH inhibits pro-inflammatory mediators in TNF-α-stimulated ARPE-19 cells by inhibiting the NF-κB/MAPK signaling pathway. Fucoidan had stronger anti-inflammatory effects than AB_SH in broad outlines and may be a potential anti-inflammatory compound for ARPE-19 cells. These results warrant further study of the AB_SH, which contains high concentrations of fucoidan underlying the biological activity of hydrothermal extracts from *S. horneri*, and it may be a good functional ingredient for managing the health of ocular disorders.

## Data availability statement

The original contributions presented in the study are included in the article/Supplementary material, further inquiries can be directed to the corresponding author/s.

## Author contributions

SL and WY: conceptualization, methodology, investigation, data curation, visualization, formal analysis, writing – original draft, and preparation. EL: data curation, visualization, and formal analysis. GL and J-HY: validation and formal analysis. WY: supervision and funding acquisition. All authors approved the submission of the manuscript.

## Footnotes

1
www.foodsafetykorea.go.kr/foodcode/01_01.jsp
