# Supplementary material for: Inhibitory effect of fucoidan on TNF-α-induced inflammation in human retinal pigment epithelium cells
Source: Front Nutr. 2023 Apr 12;10:1162934. doi: 10.3389/fnut.2023.1162934 (PMC10130517; doi:10.3389/fnut.2023.1162934)
Supplement: Supplementary file 1 [file Table_1.docx]

| **Primer** | **Sequence** |
| --- | --- |
| Human 18s rRNA | F: 5’-ATCACCATTATGCAGAATCCACG-3’ |
|  | R: 5’-GACCTGGCTGTATTTTCCATCC-3’ |
| Human IL-6 | F: 5’-TGCAATAACCACCCCTGACC-3’ |
|  | R: 5’-GTGCCCATGCTACATTTGCC-3’ |
| Human IL-1β | F: 5’-GGGACAGGATATGGAGCAACA-3’ |
|  | R: 5’-TTTCAACACGCAGGACAGGTA-3’ |
| Human TNF-α | F: 5’-TGGCGTGGAGCTGAGAGATAA-3’ |
|  | R: 5’-TTGATGGCAGAGAGGAGGTTGA-3’ |
| Human IL-8 | F: 5’-TTTTGCCAAGGAGTGCTAAA-3’ |
|  | R: 5’-CTCTGCACCCAGTTTTCCTT-3’ |
| Human MCP-1 | F: 5’-CCCCAGTCACCTGCTGTTAT-3’ |
|  | R: 5’-GCTTCTTTGGGACACTTGCT-3’ |

Supplementary Table 1. Sequences of the primers used for real time RT-PCR
